# Acute HCV/HIV Coinfection Is Associated with Cognitive Dysfunction and Cerebral Metabolite Disturbance, but Not Increased Microglial Cell Activation

**DOI:** 10.1371/journal.pone.0038980

**Published:** 2012-07-12

**Authors:** Lucy J. Garvey, Nicola Pavese, Anil Ramlackhansingh, Emma Thomson, Joanna M. Allsop, Marios Politis, Ranjababu Kulasegaram, Janice Main, David J. Brooks, Simon D. Taylor-Robinson, Alan Winston

**Affiliations:** 1 Department of Medicine, Imperial College London, London, United Kingdom; 2 Department of HIV and GU Medicine, Imperial College Healthcare NHS Trust, London, United Kingdom; 3 Neurosciences Section, Division of Experimental Medicine, Imperial College London, London, United Kingdom; 4 Imaging Sciences Department, Medical Research Council (MRC) Clinical Sciences Centre, Imperial College London, London, United Kingdom; 5 Department of HIV and GU Medicine, Guy’s and St Thomas’ NHS Foundation Trust, London, United Kingdom; Centre Hospitalier Universitaire Vaudois Lausanne - CHUV, UNIL, Switzerland

## Abstract

**Background:**

Microglial cell activation and cerebral function impairment are described in both chronic hepatitis C viral (HCV) and Human-Immune-Deficiency viral (HIV) infections. The aim of this study was to investigate the effect of acute HCV infection upon cerebral function and microglial cell activation in HIV-infected individuals.

**Methods:**

A case-control study was conducted. Subjects with acute HCV and chronic HIV coinfection (*aHCV*) were compared to matched controls with chronic HIV monoinfection *(HIVmono)*. *aHCV* was defined as a new positive plasma HCV RNA within 12 months of a negative RNA test. Subjects underwent neuro-cognitive testing (NCT), cerebral proton magnetic resonance spectroscopy (^1^H-MRS) and positron emission tomography (PET) using a ^11^C-radiolabeled ligand (PK11195), which is highly specific for translocator protein 18 kDA receptors on activated microglial cells. Differences between cases and controls were assessed using linear regression modelling.

**Results:**

Twenty-four *aHCV* cases completed NCT and ^1^H-MRS, 8 underwent PET. Of 57 *HIVmono* controls completing NCT, 12 underwent ^1^H-MRS and 8 PET. Subjects with *aHCV* demonstrated on NCT, significantly poorer executive function (mean (SD) error rate 26.50(17.87) versus 19.09(8.12), *p* = 0.001) and on ^1^H-MRS increased myo-inositol/creatine ratios (mI/Cr, a marker of cerebral inflammation) in the basal ganglia (ratio of 0.71(0.22) versus 0.55(0.23), *p* = 0.03), compared to subjects with *HIVmono*. On PET imaging, no difference in ^11^C-PK11195 binding potential (BP) was observed between study groups (*p*>0.10 all cerebral locations), however lower BPs were associated with combination antiretroviral therapy (cART) use in the parietal (*p* = 0.01) and frontal (*p* = 0.03) cerebral locations.

**Discussion:**

Poorer cognitive performance and disturbance of cerebral metabolites are observed in subjects with *aHC,V* compared to subjects with *HIVmono*. Higher ^11^C-PK11195 BP was not observed in subjects with *aHCV*, but was observed in subjects not on cART.

## Introduction

Central nervous system disturbances are well described in patients with chronic HIV infection and more recently, in those with chronic hepatitis C (HCV) infection in the absence of significant liver disease[1]–[3]. Abnormalities, compared to matched control populations, demonstrable using cerebral function measures such as neurocognitive testing (NCT) and proton magnetic resonance spectroscopy (^1^H MRS) have been shown in both patient groups [4], [5]. Such cerebral deficits may be greater in individuals coinfected with HIV and chronic HCV, compared to individuals with HIV monoinfection [6]–[9]. Both viruses can be identified in cerebrospinal fluid and brain tissue [9]–[11] and although the pathophysiology of these cerebral disturbances remain unclear, microglial cell activation has been proposed as a promoter of neurodegeneration and inflammation in both HIV-associated encephalopathy [12] and chronic HCV-associated cognitive decline [13].

The isoquinoline PK11195 is a highly specific, high-affinity ligand for translocator protein 18 kDA receptors on activated microglial cells. PK11195 can be radio-labeled with carbon 11 (^11^C) and injected as a tracer during cerebral positron emission tomography (PET) scanning to provide non-invasive, quantification of microglial cell activity at a cellular level. This technique has been histologically validated in animals [14], [15] and humans [16] and used to demonstrate microglial activation in a variety of human neurological disorders, including in patients with chronic HCV in the presence or absence of cirrhosis [17]–[20]. To date, increased binding of PK11195 has been demonstrated only in HIV-infected subjects with severe HIV encephalopathy [21], with increased binding not reported in asymptomatic HIV-infected subjects or in those with mild cognitive deficits [22].

The impact of acute HCV infection acquisition upon cerebral function parameters remains poorly understood. Secondary to a recent epidemic of acute HCV infection in HIV-infected men who have sex with men, this phase of infection is now frequently identified in clinical practice [23], [24]. Earlier work from our group [25] identified a reduction in inflammatory cerebral metabolites in the basal ganglia of 10 subjects with acute HCV and chronic HIV infection. The aim of this study, was to further elucidate any changes in cerebral function observed in subjects during acute HCV infection via the assessment of cognitive performance and cerebral metabolites and to assess for any evidence of increased microglial cell activation via PET imaging.

## Methods

Ethics approval was obtained from the National Research Ethics Service in separate applications to undertake the NCT studies (07/H0803/128) and ^1^H MRS (08/H0712/15). For the administration of ^11^C PK11195 (09/H0712/17) permission was also obtained from the Administration of Radioactive Substances Advisory Committee (ARSAC) of the UK**.** All participants provided written, informed consent prior to commencing any study procedures.

All subjects completed NCT studies with a sub-set enrolled in ^1^H-MRS and a smaller group undergoing PET examination. All subjects were recruited during routine HIV clinic attendances and were eligible if aged over 18 years with chronic HIV infection (HIV-1 antibody positive for a minimum of 6 months). Exclusion criteria included current AIDS-defining illnesses, any active neurological complaint or disease, encephalopathy, untreated syphilis, chronic hepatitis B infection, current receipt of interferon and ribavirin treatment, hepatic synthetic functional impairment (a serum albumin below 30 g/dL or prolonged prothrombin time), use of benzodiazepines or recreational drugs within the past month and alcohol consumption in excess of an average of 20 g/day in the past six months.

### Selection of Subjects with Acute Hepatitis C

Subjects with HIV/acute HCV coinfection *(aHCV cases)* were required to have acute HCV, defined by a new positive plasma HCV RNA test within 12 months of a negative HCV RNA test.

### Selection of Control Subjects

HIV-infected individuals without hepatitis C co-infection (*HIVmono*), were recruited as control subjects. All were required to be HCV antibody or RNA negative within the past year, with normal liver function tests thereafter. For participants undergoing ^1^H-MRS and PET, subjects were matched to *aHCV cases* according to age, time-elapsed since HIV diagnosis, current CD4+ cell count, whether currently receiving combination antiretroviral therapy (cART), and type of cART in those receiving therapy (non-nucleoside reverse transcriptase inhibitor (NNRTI)-based or protease inhibitor (PI)-based).

### Neurocognitive Test (NCT)

A computerised assessment of cognitive performance was undertaken (*CogState™, Melbourne, Australia*) which has been previously validated in HIV infection [26] and used in HIV treatment studies [27]. It assesses 8 cognitive domains (psychomotor function, identification, monitoring and matched learning; associate learning, visual learning and working memory; and executive function). Overall cognitive speed, performance accuracy, executive function and composite *Z*-scores were then calculated for each subject.

### Proton Magnetic Resonance Spectroscopy (^1^H-MRS)

^1^H-MRS was performed on an Achieva™ 1.5 Tesla scanner (*Phillips NV, Best, Netherlands*) at the Robert Steiner Magnetic Resonance Unit, Hammersmith Hospital, London, UK. Examination included sagittal, coronal and axial T_1_-weighted images of the brain and T_2_-weighted axial double spin echo images. ^1^H-MRS was performed in 3 voxel locations: frontal white matter (FWM), frontal grey matter (FGM) and the basal ganglia (BG), using a double spin echo point resolved spectroscopy (PRESS) sequence with the following settings: echo time (TE) 36 ms, repetition time (TR) 3000 ms, 2048 data points, spectral width of 2500 Hz and 128 data acquisitions. MR spectra were post-processed for automated water signal suppression and water shimming. T_1_ and T_2_-weighted MR images were studied by a neuroradiologist. All spectra were analysed and quantified by one observer (LG) using a java-based version of the magnetic resonance user interface package (jMRUI Version Number: 3.0) [28], incorporating the AMARES algorithm [29] and metabolites expressed as ratios to cerebral creatine (Cr).

### Cerebral PET with ^11^C Labelled PK11195

For subjects undergoing PET scanning, a strict 1∶1 matching process of cases to controls was applied according to the selection of subjects above using the following criteria for matching; all gender matched, age (within 5 years), elapsed time since HIV diagnosis (within 5 years), current CD4+ count (within 100 cells/uL), nadir CD4+ count (within 100 cells/uL), whether currently receiving cART and type of cART (NNRTI or PI-based regimens matched).

PET scanning was performed on a PET-CT scanner (Whole body positron emission tomograph GE Discovery Rx PET/CT, *GE Healthcare, Waukesha, Wisconsin*) at the MRC Cyclotron Building, Hammersmith Hospital, Imperial College, London. A transmission CT scan and emission scan were performed with subjects lying in a partially-enclosed PET scanner and an injection of ^11^C-labeled PK11195 radioactive ligand [R-enantiomer] was given as a smooth bolus. The target quantity was 296 MBq (8.00 mCi, approximately 1.7mSv tissue dose). Parametric images of specific ^11^C –[R]-PK11195 binding potential (BP), a measure reflecting Bmax/Kd, were calculated using a basis function implementation of a simplified reference tissue model [30] **.** The possible widespread expression of TSPO in patients with viral infections makes it difficult to select a reference region representing nonspecific PK uptake in the brain. We therefore used a supervised clustering procedure to identify reference clusters of voxels in the grey matter having PK11195 time activity curves mirroring those of a standardized control population, as previously described [31].

For each patient, PK11195 BP values in the parietal, occipital, frontal, temporal, ventral striatum, caudate, putamen and thalamus regions were calculated by applying standardised object maps to normalized ^11^C-[R]-PK11195 BP images in Analyze software (Analyze AVW, Mayo Clinic, US). Spatial normalization of parametric images into standard stereotaxic space (Montreal Neurologic Institute, MNI) was achieved by normalizing the individual MRI T1 image to the T1 image template in MNI space available in SPM5 software (http://www.fil.ion.ucl.ac.uk/spm/software/spm5/) and then applying the transformation parameters to the respective BP images previously co-registered to the individual MRI T1 image.

### Statistical Analysis

Univariate linear regression analysis was used to investigate the presence of association between cerebral function assessments in each study (NCT scores, ^1^H-MRS cerebral metabolite ratios and PK11195 BP) and study group. Where significant differences were found, linear regression analysis was used to investigate association between assessment results and clinical parameters. Any significant baseline parameter discrepancy between groups or associated factors with a significance of p<0.15 were taken forward to multivariate analysis. SPSS version 18.0 was used for analysis. *p*-values of <0.05 were considered statistically significant.

## Results

### Baseline Characteristics

Overall, 81 subjects completed NCT procedures with baseline characteristics described in *Table 1* *.* A significantly lower proportion of the *aHCV* group were receiving cART than in the *HIVmono* group (71% versus 95% respectively, *p* = 0.006). All *aHCV* subjects had detectable HCV viraemia and elevated transaminases at the time of study entry and had a documented, previously negative HCV RNA test within a median of 24 weeks (range 4–48). No subject had impaired hepatic synthetic function or hepatic failure.

**Table 1:** Patient demographics, clinical parameters and study participation of subjects completing neurocognitive testing (NCT) procedures.

StudyParticipation	Clinical parameter	Acute HCV/HIV*(aHCV)*	HIV monoinfection*(HIVmono)*	Difference betweengroups, *p*-value*
**Overall**	**Number,** *n*	24	57	
	**Age (years),** *median [IQR]*	41 [36], [44]	47 [39, 56]	**0.003**
	**Male gender,** *n (%)*	24 (100)	50 (89)	0.10
	**Time-elapsed since HIV diagnosis (years),** *median [IQR]*	6 [3], [12]	11 [5], [16]	**0.02**
	**Current CD4+(cells/µL),** *median [IQR]*	590 [458, 745]	505 [382, 783]	0.29
	**Nadir CD4+(cells/µL),** *median [IQR]*	200 [215, 395]	205 [88, 283]	**0.01**
	**Receiving antiretroviral therapy,** *n (%)*	17 (71)	54 (95)	**0.006**
	**Current plasma HIV viral load below 50 c/mL,** *n (%)*	16 (67)†	54 (95)	**0.002**
	**HIV viral load of remaining subjects (c/mL),** *median [IQR]*	5797 [1136, 11758]	14182 [1095, 15952]	0.18
	**Time elapsed since negative HCV RNA test (weeks),** *median [IQR]*	24 [20], [32]	–	–
	**Current ALT (IU),** *median [IQR]*	213 [78, 237]	–	–
	**Peak ALT (IU),** *median [IQR]*	237 [180, 820]	–	–
	**HCV genotype 1,** *n (%)*	21 (88)	–	–
	**Most recent HCV RNA (c/mL),** *median [IQR]*	986855 [56650, 4315528]	–	–

Table 1 legend: HCV = hepatitis C virus; RNA: ribonucleic acid; ALT  =  alanine aminotransferase; IU = international units;

† 1 subject had VL 87 copies/mL at time of assessment, repeat <50 copies/mL, *using Fisher’s exact test or t-test].

### Neurocognitive Testing Results

No significant differences were observed in overall composite *Z*-score or performance accuracy between study groups (p≥0.05 all observations, *Table 2*). However individuals with *aHCV* had significantly poorer executive function performance than *HIVmono* in the univariate analysis (p = 0.02, 95%CI 0.09, 1.07). When factors associated with poorer executive function were examined in a multivariate model, no association with receiving cART was observed (*p* = 0.37, 95%CI −18.8, 7.1), however lower nadir CD4+ cell count and *aHCV* study group were independently associated with poorer executive function performance (*Table 3*). In the univariate analysis, *aHCV* was associated with faster cognitive speed, but in the multivariate analysis, after adjustment for age, cART status, nadir and current CD4+ cell count, this association was no longer statistically significant (*Table 3*).

**Table 2:** Results of cerebral function assessments and univariate regression analysis to investigate differences between subject groups.

Cerebral functionassessment,*mean (SD)*			AcuteHCV/HIV*(aHCV)*	HIVmonoinfection*(HIVmono*)	*p*-value	[95%CI]
**NCT**	**Number of participants, ** ***n***		**24**	**57**		
	**Participants on cART, n(%)**		**17 (71%)**	**54 (95%)**		
	**Cognitive speed (logms)**		**10.57 (0.28)**	**10.73 (0.33)**	**0.05**	**[**−**0.99, 0.01]**
	**Accuracy (arc.proportion correct)**		3.00 (0.26)	2.88 (0.42)	0.20	[−0.18, 0.83]
	**Executive function (error rate)**		**26.50 (17.87)**	**19.09 (8.12)**	**0.02**	**[0.09, 1.07]**
	**Composite ** ***Z*** **-score**		0.16 (2.27)	−0.08 (2.25)	0.68	[−0.90, 1.38]
**1H-MRS**	**Number of participants, ** ***n***		24	12		
	**Participants on cART, n(%)**		17 (71%)	9 (75%)		
	**Frontal grey matter**	*NAA/Cr*	1.42 (0.25)	1.35 (0.10)	0.32	[−0.41, 1.21]
		*Cho/Cr*	0.59 (0.12)	0.63 (0.17)	0.31	[−2.38, 0.77]
		*mI/Cr*	0.70 (0.28)	0.62 (0.13)	0.39	[−0.10, 0.25]
	**Frontal white matter**	*NAA/Cr*	1.53 (0.32)	1.48 (0.26)	0.63	[−0.42, 0.68]
		*Cho/Cr*	1.04 (0.20)	1.02 (0.19)	0.73	[−0.69, 0.98]
		*mI/Cr*	0.96 (0.48)	0.83 (0.54)	0.47	[−0.23, 0.49]
	**Basal ganglia**	*NAA/Cr*	1.71 (0.25)	1.65 (0.29)	0.54	[−0.45, 0.84]
		*Cho/Cr*	0.77 (0.12)	0.80 (0.19)	0.36	[−2.27, 0.85]
		*mI/Cr*	0.71 (0.22)	0.55 (0.23)	0.06	[−0.01, 0.32]
**PK11195 BP**	**Number of participants, ** ***n***		8	8		
	**Participants on cART, n(%)**		6 (75%)	6 (75%)		
	**Ventral striatum**		0.18 (0.10)	0.22 (0.13)	0.45	[−3.46, 1.64]
	**Caudate**		0.08 (0.04)	0.09 (0.08)	0.69	[−5.76, 3.94]
	**Putamen**		0.21 (0.11)	0.28 (0.14)	0.29	[−3.49, 1.14]
	**Thalamus**		0.52 (0.31)	0.55 (0.17)	0.84	[−1.44, 1.19]
	**Parietal**		0.07 (0.06)	0.10 (0.06)	0.33	[−7.56, 2.71]
	**Temporal**		0.13 (0.03)	0.16 (0.05)	0.16	[−10.82, 1.99]
	**Occipital**		0.13 (0.04)	0.19 (0.09)	0.10	[−6.58, 0.54]
	**Frontal**		0.83 (0.40)	1.03 (0.27)	0.26	[−1.34, 0.40]

[Legend Table 2: *FGM =  frontal grey matter; FWM = frontal white matter; BG = basal ganglia; NAA = N-acetyl aspartate; Cr = creatine; Cho = choline; mi = myo-inositol peak*].

**Table 3:** Results of univariate and multivariate analyses to examine clinical parameters associated with executive function performance, composite speed and basal ganglia mI/Cr ratio.

Cerebral functionassessment result	Poorerexecutivefunction	Poorerexecutivefunction	FasterCompositespeed	FasterCompositespeed	Higher basalgangliamI/Cr ratio	Higher basalgangliamI/Cr ratio
	Univariate	Multivariate	Univariate	Multivariate	Univariate	Multivariate
***Clinical parameter***	***p*** **-value** [**95% CI]**	***p-*** **value [95% CI]**	***p*** **-value [95% CI]**	***p-*** **value [95% CI]**	***p*** **-value [95% CI]**	***p-*** **value [95% CI]**
***aHCV*** ** study group**	**0.02 [0.09, 1.07]**	**0.001 [5.8, 20.1]**	**0.05 [**−**0.99, 0.00]**	**0.22 [**−**0.3, 0.1]**	0.06 [−0.01, 0.32]	**0.03 [0.02, 0.35]**
**Nadir CD4+ count**,*per 100 cell/uL increase*	0.09 [−0.30, 0.02]	**0.001 [**−**0.1,** −**0.02]**	**0.03 [**−**0.33,** −**0.02]**	**0.10 [**−**0.01, 0.00]**	0.09 [−0.10, 0.01]	**0.05 [**−**0.11, 0.00]**
**Current CD4+ count**,*per 100 cell/uL increase*	0.70 [−0.12, 0.08]	−	**0.05 [**−**0.20,** −**0.00]**	0.78 [−0.05, 0.03]	0.83 [−0.05, 0.04]	−
**Age**, *per 10 year increase*	0.68 [−0.28, 0.18]	0.59 [−3.9, 2.2]	**0.03 [0.02, 0.47]**	0.20 [−0.30, 1.30]	0.45 [−0.12, 0.06]	−
**Receiving cART**	0.70 [−0.95, 0.65	0.37 [−18.8, 7.1]	0.42 [−0.47, 1.12]	0.79 [−0.34, 0.26]	0.58 [−0.14, 0.24]	−
**Plasma VL**,*per 1000 c/mL increase*	0.37 [−0.38, 0.14]	0.06 [−0.54, 0.01]	0.82 [−0.01, 0.01]	0.59 [−0.01, 0.01]	0.84 [−0.01, 0.01]	−
**Time since HIV diagnosis**,*per 10 year increase*	0.34 [−0.06, 0.02]	0.15 [−0.85, 0.13]	0.57 [−0.03, 0.05]	0.54 [−0.02, 0.01]	0.81 [−1.58, 1.25]	−

### 1H-MRS

Twenty-four *aHCV* subjects and 12-matched *HIVmono* subjects also underwent cerebral ^1^H-MRS. Subjects were matched according to age, gender and cART status. All 36 participants were male. Mean(SD) age and proportion receiving cART for *aHCV* and *HIVmono* were 40(8) versus 44(12) years and 71% versus 75% respectively. The mean (SD) time elapsed since last negative plasma HCV RNA in the *aHCV* patients prior to ^1^H-MRS was 24 weeks.

Cerebral metabolite ratios demonstrated a trend towards higher mI/Cr ratios in the basal ganglia (BG) of *aHCV* subjects (*p* = 0.06, 95% CI for difference between groups −0.01, 0.32, see *Table 2*). In a multivariate model, both nadir CD4 count and *aHCV* study group were independently associated with higher mI/Cr (p = 0.05 and p = 0.03 respectively, *Table 3*). No significant associations were observed between BG mI/Cr and clinical parameters including the time elapsed since HCV RNA negative test, age or current CD4+ count (p-value>0.45 all values). Of note in subjects with *aHCV*, a strong association between higher BG mI/Cr and poorer executive function was observed (p = 0.006, 95%CI 0.00, 0.01).

### Cerebral PET with ^11^C Labelled PK11195

16 subjects (8 in each group) then underwent a PET examination with all cases and controls matched for gender (all male) and HIV disease characteristics; numbers receiving no cART, NNRTI-based and boosted PI-based cART were 2, 4 and 2 subjects in each group, respectively. The mean number of weeks elapsed since most recent negative plasma HCV RNA test in this smaller aHCV sub-group was 20.5 (range 16–28) indicating very recent acquisition in all subjects.

**Figure 1:**
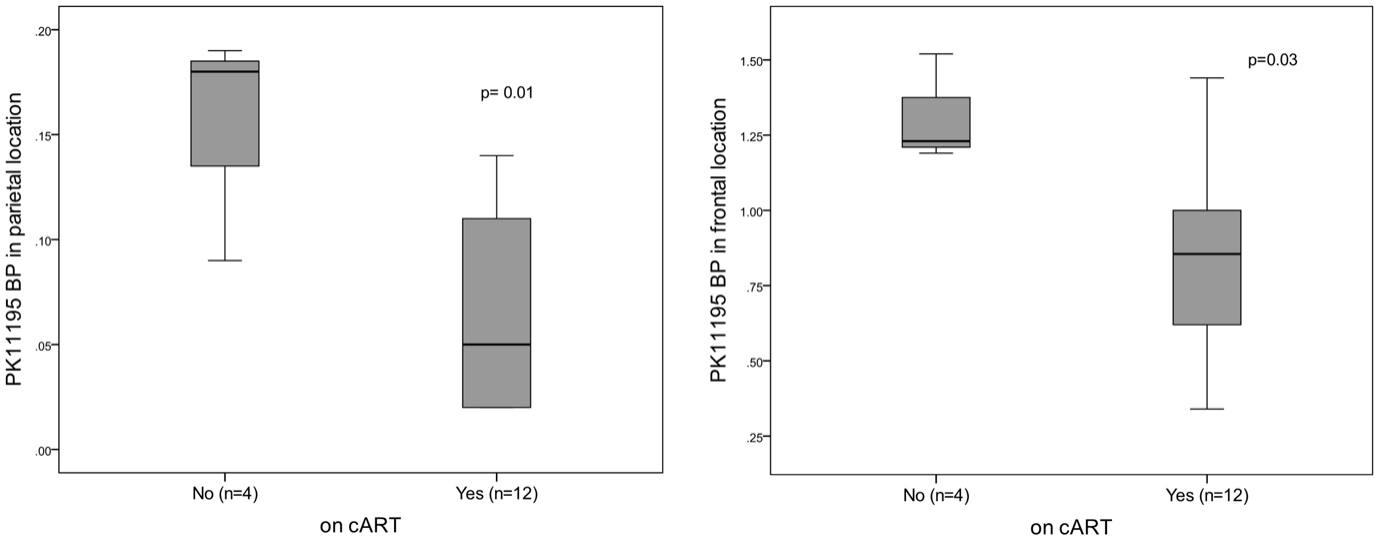
Box-plots to demonstrate PK11195 Binding Potential (BP) in parietal and frontal locations according to Combination Antiretroviral therapy (cART) status.

The reference regions were within the cortex and no patterns were observed in their extent/location. No overlap with reference regions and regions of interest occurred. Overall PK11195 BPs for each cerebral region of interest are shown in *Table 2*. No significant difference between *aHCV* cases and controls was observed in any location (p>0.10 all values), but in the multivariate analysis, receiving cART was independently associated with significantly lower PK11195 BP in the frontal (p = 0.05, 95% CI −0.82, 0.02) and parietal locations (p = 0.03, 95%CI −0.14, −0.01), *see* *Table 4* *and* *Figure 1*).

**Table 4:** Results of univariate and multivariate analyses to examine clinical parameters associated with occipital, parietal and frontal PK11195 Binding Potential (BP).

Cerebral locationof PK11195binding Potential (BP)	Occipital	Occipital	Parietal	Parietal	Frontal	Frontal
	Univariate	Multivariate	Univariate	Multivariate	Univariate	Multivariate
***Clinical parameter***	***p*** **-value** [**95% CI]**	***p-*** **value [95% CI]**	***p*** **-value [95% CI]**	***p-*** **value [95% CI]**	***p*** **-value [95% CI]**	***p-*** **value [95% CI]**
***aHCV*** ** study group**	0.10 [−0.15, 0.01]	0.11 [−0.13, 0.02]	0.33 [−0.10, 0.03]	−	0.26 [−0.58, 0.17]	−
**Nadir CD4+ count**,*per 100 cell/uL increase*	0.65 [−0.02, 0.03]	−	0.18 [−0.01, 0.03]	−	0.24 [−0.04, 0.14]	−
**Current CD4+ count**,*per 100 cell/uL increase*	0.68 [−0.02, 0.03]	−	0.06 [−0.01, 0.03]	0.14 [−0.01, 0.02]	0.13 [−0.02, 0.17]	0.29 [−0.04, 0.14]
**Age**, *per 10 year increase*	0.62 [−0.05, 0.03]	−	0.48 [−0.04, 0.02]	−	0.35 [−0.87, −0.07]	−
**Receiving cART**	0.06 [−0.19, 0.00]	0.07 [−0.18, 0.09]	**0.01 [**−**0.15,** −**0.02]**	**0.03 [**−**0.14,** −**0.01]**	**0.03 [**−**0.87,** −**0.07]**	**0.05 [**−**0.82, 0.02]**

## Discussion

In this study, we have demonstrated that acquisition of acute HCV in HIV-infected subjects is associated with changes in cerebral function measures using a cognitive assessment and cerebral ^1^H-MRS. We did not however, demonstrate significant differences in PK11195 BP when using PET imaging as an *in vivo* marker of microglial cell activation.

Acute HCV was independently associated with poorer executive function performance in this study, despite the relatively well-preserved CD4+ cell counts and young age of subjects enrolled. Importantly, these differences remained statistically significant after adjustment for the lower proportion of *aHCV* subjects receiving cART. While this form of higher cognitive processing has previously been described in HIV and chronic HCV/HIV coinfection [6], [32], it has not previously been associated with the acute phase of HCV. These findings suggest that a neurological disturbance involving the prefrontal cortex and fronto-striatal regions may occur, in a similar manner to chronic HIV infection [33], [34]. Aside from nadir CD4+ cell count, no association with executive function and other clinical parameters were found as an alternative explanation, supporting acute HCV viraemia as the potential cause of this deficit. Unfortunately, without cognitive assessment data which precedes HCV acquisition, it is not possible to confirm these findings reflect an acute deterioration in cognitive performance associated with the acquisition of HCV infection.

Statistically significantly greater mI/Cr ratios were observed in the BG of subjects with acute HCV, when compared to matched controls in our multivariate model. Proportions of subjects receiving cART were very similar in both groups. mI is an osmosensitive glial marker and plays a crucial role in cell volume regulation [35]. The changes to mI/Cr ratio that were observed may represent increased neuroinflammation and glial proliferation during the early phase of HCV. Forton *et al* [36] have previously described increases of mI/Cr ratio in patients with chronic HCV, in the frontal white matter, rather than the BG. As the BG has a higher blood flow per unit volume [37] than other cerebral locations, it is possible that greater and earlier exposure of this cerebral location may explain why the changes observed in our study during acute infection had not yet evolved in other areas of the brain. Furthermore, BG dysfunction is associated with symptoms of fatigue and inertia in other neurological disorders and therefore inflammatory changes in this location, may explain the cognitive deficits we observed [38]. Interestingly and in contrast, in our earlier pilot study, we reported reductions in BG mI/Cr ratios in 10 subjects with acute HCV [25]. The changes we now report within a larger cohort may reflect a time dependent phenomena whereby dynamic changes in mI/Cr ratios are occurring during the early course of acute HCV infection. In our previous work, an average of 16 weeks had elapsed since most recent negative HCV RNA result, suggesting very recent acquisition of HCV virus. In this larger MRS study, the mean time elapsed since last negative HCV RNA result was 24 weeks and therefore we postulate these osmosensitive glial markers may change rapidly during acute HCV infection. Alternatively, other factors such as the small size of the earlier pilot study or differences in HIV and cART parameters of the participants may be responsible for these apparently contradictory findings.

No differences in PK11195 BP were observed between our study groups. Several plausible explanations for these findings exist. First, microglial cells may not be activated by HCV *in vivo*, but an alternative cell-type may be responsible, or indeed, CNS neurological disturbance may not occur at all. Alternatively, microglial cell recruitment may only take place during later stages of chronic HCV infection, as the inflammatory process may take time to develop, given the chronic nature of HCV infection over many decades. The first theory is perhaps refuted and last theory supported by the findings of Grover and colleagues who describe increased PK11195 BP, interpreted as microglial cell activation, in patients with chronic HCV without significant liver disease and by the findings of Cagnin and colleagues who observed similarly increased PK11195 BP in a small number of subjects with advanced, chronic HCV infection using the same PET methodologies [36]. It is therefore feasible that during the acute period of infection with HCV, there is no change to levels of microglial cell activation. It is known that microglia are recruited from circulating macrophage/monocytes and animal model data have demonstrated that this process may take several months following cerebral insult [39]. The mean time elapsed since negative HCV RNA test was 21 weeks in the 8 *aHCV* subjects of this PET study, which is likely to be an over-estimation of *actual* time since viral transmission. It is therefore possible, if our study was repeated several months later, different results would be observed. Another explanation for our findings is that due to ligand-factors including high plasma-protein binding, non-specific binding or low brain uptake, PK11195 as a neurotracer was insufficiently sensitive to detect subtle changes in microglial activation in such neurologically asymptomatic subjects, and that use of other, newly available, alterative ligands including ^11^C PBR-28 [40], ^11^C DPA-713 [41] or ^11^C DAA-1106 [42] may alter our findings. Further PET studies are needed to answer these questions. If microglial activation is a feature of the chronic phase of HCV infection, we postulate that the effects of circulating cytokines (rather than microglial cell activation) may be responsible for the cerebral function differences we observed in this study. The neuropsychological effects of endogenous circulating cytokines and chemokines are observed in the presence of chronic infection and are very similar to the CNS effects of therapeutically-administered interferon.

Interestingly, we observed an association between those receiving cART and lower PK11195 BP values in some cerebral locations, which has not been previously described and which suggests lower levels of microglial cell activation in subjects receiving cART, irrespective of acute HCV status. While only small numbers of patients were sampled and few number of subjects were not receiving cART, this suggests that, similar to systemic levels of inflammation, neuroinflammation may also be reduced in effectively treated HIV-infected subjects. Over time, ongoing inflammation within the CNS may result in progressive neuronal damage with subsequent clinical sequelae and these data suggest an additional potential benefit from cART, which although may be postulated, have not to date been clearly demonstrated.

Associations between recreational drug use, concurrent sexually-transmitted infections and acquisition of acute HCV have been described which may influence cerebral metabolite ratios and have a negative impact upon cognitive performance [43]. For this reason, any subject with recent recreational drug misuse (within past 4 weeks) or current sexually transmitted infection known to cause CNS disease (such as early syphilis) were excluded. It remains possible, however, that high rates of historical drug misuse (with resultant cerebral damage) may have contributed to our observations. A further limitation of this work is the absence of longitudinal follow-up studies to identify if any increase of ^11^C PK11195 BP evolves with duration of HCV coinfection, although it should be noted that ethical considerations on radiation dosage has precluded the evaluation of such longitudinal PET studies to date. Finally, the sample size for the ^1^H-MRS and PET imaging studies, limits our ability to detect subtle differences and associations. PET scans with PK11195 are too costly for large-scale studies, but our work is of similar size to previous physiological PET studies and larger than other previously published work in this area.

In summary, we report changes to cerebral function measures in subjects with acute HCV, but have not found differences of PK 11195 BP, representing microglial cell activation, in the early months of HCV viraemia. If microglial activation is a late feature of chronic HCV infection, then there may be an additional clinical reason to treat and eliminate HCV in the acute phase where possible, as therapy-response rates are higher and recommended treatment courses shorter [44].
